# Electroacupuncture Promotes Nerve Regeneration and Functional Recovery Through Regulating lncRNA GAS5 Targeting miR-21 After Sciatic Nerve Injury

**DOI:** 10.1007/s12035-023-03613-3

**Published:** 2023-09-06

**Authors:** Ming-yue Tian, Yi-duo Yang, Wan-ting Qin, Bao-nian Liu, Fang-fang Mou, Jing Zhu, Hai-dong Guo, Shui-jin Shao

**Affiliations:** School of Integrative Medicine, Shanghai University of Traditional Chinese Medicine, Shanghai, 201203 China

**Keywords:** Electroacupuncture, Sciatic nerve injury, lncRNA GAS5, miR-21, Functional recovery, Nerve regeneration

## Abstract

Although the benefits of electroacupuncture (EA) for peripheral nerve injury (PNI) are well accepted in clinical practice, the underlying mechanism remains incompletely elucidated. In our study, we observed that EA intervention led to a reduction in the expression of the long non-coding RNA growth-arrest-specific transcript 5 (GAS5) and an increased in miR-21 levels within the injured nerve, effectively promoting functional recovery and nerve regeneration following sciatic nerve injury (SNI). In contrast, administration of adeno-associated virus expressing GAS5 (AAV-GAS5) weakened the therapeutic effect of EA. On the other hand, both silencing GAS5 and introducing a miR-21 mimic prominently enhanced the proliferation activity and migration ability of Schwann cells (SCs), while also inhibiting SCs apoptosis. On the contrary, inhibition of SCs apoptosis was found to be mediated by miR-21. Additionally, overexpression of GAS5 counteracted the effects of the miR-21 mimic on SCs. Moreover, SCs that transfected with the miR-21 mimic promoted neurite growth in hypoxia/reoxygenation-induced neurons, which might be prevented by overexpressing GAS5. Furthermore, GAS5 was found to be widely distributed in the cytoplasm and was negatively regulated by miR-21. Consequently, the targeting of GAS5 by miR-21 represents a potential mechanism through which EA enhances reinnervation and functional restoration following SNI. Mechanistically, the GAS5/miR-21 axis can modulate the proliferation, migration, and apoptosis of SCs while potentially influencing the neurite growth of neurons.

## Introduction

Approximately five million cases of peripheral nerve injury (PNI) annually occur in the USA alone, requiring over $1.5 billion in nerve reconstruction treatments [1]. Despite the relatively robust regenerative capacity of the adult peripheral nervous system (PNS) compared to the central nervous system, the outcomes of nerve regeneration and functional recovery remain unsatisfactory [2]. Autogenous nerve grafting currently serves as the gold standard in the treatment for PNI [3]; however, the insufficient donor nerve source is the major limitation [4]. Electroacupuncture (EA), a therapeutic modality involving the electrical stimulation of specific acupoints on the body, has a long-standing history of application in the Eastern world for treating various disorders [5]. Although the clinical benefits of EA in PNI are widely acknowledged [6, 7], the underlying mechanism remains incompletely understood.

MicroRNAs (miRNAs) are short RNA molecules, approximately 21 nucleotides in length, that play a crucial role in posttranscriptional gene regulation [8]. They are involved in a wide range of physiological processes and pathological conditions [9, 10]. Emerging evidence indicates that miRNAs play a significant role in recovery process after nerve injury. Among these miRNAs, miR-21 has been extensively investigated and has been found to possess multiple critical regulatory functions. For example, miR-21 has been shown to increase in response to spinal cord injury (SCI) and exerts various protective effects against SCI [11]. Notably, our previous study also demonstrated that the overexpressed miR-21 promoted neurite outgrowth [12]. Most importantly, miR-21 has been implicated in the mechanism through which EA promotes functional recovery and nerve regeneration after sciatic nerve injury (SNI) [13]. However, the precise mechanisms underlying the upregulation of miR-21 induced by EA after PNI remain unknown.

Long non-coding RNAs (lncRNAs) are transcripts that are longer than 200 nucleotides, generally without protein-coding potential [14]. Recently researchers have suggested that lncRNAs may function as ceRNAs that compete with miRNAs for binding sites [15, 16]. The role of GAS5, as a lncRNA, in the repair of brain injury has been widely reported [17]. Several researchers have reported that GAS5 acts as an inhibitory factor in axon regeneration [18, 19]. Specifically, the knockdown of GAS5 has been shown to enhance neurite growth in dorsal root ganglion (DRG) neurons in rats [20]. Consistently, GAS5 knockout mice exhibited enhanced nerve regeneration capacity following SNI [20]. However, the potential role of GAS5 in the context of EA-mediated promotion of repair in SNI remains to be further elucidated. Additionally, previous studies have indicated the presence of a complementary region between GAS5 and miR-21, suggesting a potential regulatory relationship [21]. Thus, we hypothesized that GAS5 may regulate the repair process after PNI by targeting miR-21.

SCs are the principal glial cells in PNS and form a myelin sheath to provide metabolic and nutritional support to neuronal axons [22]. In addition, SCs are involved in guiding neurite growth through their migration and proliferation [23], and they contribute to the clearance of axon and myelin debris after PNI [24]. However, the specific mechanisms by which GAS5 regulates the SCs proliferation, migration, and apoptosis of SCs in vitro remain unclear. To address this, we utilized the NG108-15 cell line, which is a hybrid cell line derived from the fusion of mouse neuroblastoma cells with rat glioma cells [25]. This cell line retains the essential biological characteristics of neuronal cells [26] and serves as a valuable tool for simulating the physiological and pathological conditions of neuronal cells [27]. In our study, we observed the differential expressions of GAS5 and miR-21 after EA intervention following PNI and aimed to determine the effect of EA and its potential mechanism in regulating GAS5. Specifically, our findings suggest that GAS5, by targeting miR-21, has the potential to promote the proliferation and migration of SCs while inhibiting apoptosis. Furthermore, GAS5 may also contribute to the neurite growth of NG108-15 cells.

## Materials and Methods

### Animals

Male SPF-grade Wistar rats weighing approximately 200 g were purchased from the Shanghai Slack Laboratory Animal Co Ltd (Shanghai, China). The rats were housed in the Laboratory Animal Center of Shanghai University of Traditional Chinese Medicine (TCM) under controlled conditions, including a 12-h light/dark cycle, a temperature of 22 ± 2 °C, and libitum access to food and water. All experimental procedures were performed following the guidelines of the National Institutes of Health Guide for the Care and Use of Laboratory Animals (revised in 1978). All surgical and experimental procedures on rats were approved by the Animal Ethics Committee, Shanghai University of TCM (PZSHUTCM211129006).

### Establishment of Sciatic Nerve Injury Model

Thirty rats were randomly assigned to three groups: the sham group, the model group, and the EA group, with each group comprising 10 rats. The SNI was employed as a commonly used model of PNI [28]. Briefly, all rats were intraperitoneally anesthetized with sodium pentobarbital at a dose of 50 mg/kg. The right lower limb of the rats was disinfected, and a posterior lateral thigh incision was made to expose the sciatic nerve. Following the sciatic nerve incision, the sciatic nerve epineuria and the skin were sutured separately using 9–0 and 3–0 silk threads under a stereomicroscope, except for the rats in the sham group.

### AAV-GAS5 Administration

Forty rats were randomly allocated into four groups: the model group, EA group, EA + AAV-GAS5 group, and EA + AAV-negative control (NC) group, with each group consisting of 10 rats. The SNI model was established as described earlier in each group. Adeno-associated virus 9 carrying GAS5 (AAV9-GAS5, AAV-GAS5) (6.5 × 10^11^ viral particles/mL) or a scramble control construct (AAV9 Scramble, AAV-NC) (BrainVTA, Wuhan, China) was injected into the site of the sciatic nerve injury at a dosage of 10 μL for the EA + AAV-GAS5 group and EA + AAV-NC group.

### EA Intervention

In the first experiment, on the second day following the establishment of the model, rats in the EA group underwent EA intervention. EA stimulation was administered for a duration of 20 min per day, targeting the “Huantiao” (GB30) and “Zusanli” (ST36) acupoints. The 0.25 × 13 mm needles were inserted into these acupoints, and the G6805A electroacupuncture apparatus was utilized to connect the needles. The positive pole and the negative pole were separately attached to GB30 and ST36, delivering an intermittent wave that induced slight muscle vibration. The treatment continued for a period of 3 weeks, with a suspension after continuous treatment for 6 days.

### Sciatic Nerve Conduction Velocity Recovery Rate (NCV)

The NCV was assessed in the sciatic nerves as previously mentioned [13]. To detect NCV, the ARM6240 biological signal acquisition system from Chengdu Instrument Factory was utilized. Electrical stimulation was induced by a stimulating electrode connected to the proximal end of the sciatic nerve. The compound motor action potentials in the distal sciatic nerve were tested using a receiving electrode, to which the acupuncture needles were attached. Subsequently, sciatic nerve conduction velocity was calculated by determining the distance/time between the stimulating electrode and the receiving electrodes. The NCV of the sciatic nerve was then evaluated by calculating the ipsilateral NCV to the contralateral NCV.

### Sciatic Nerve Function Index (SFI)

A rectangular box was prepared, and the bottom was covered with white A4 paper. Once the hind paws of the rats were fully stained with ink, they were allowed to walk freely within the box. The footprints left by the rats were then utilized for the analysis of the SFI. In this analysis, PL referred to the distance from the heel to the toe, TS represented the width between toes 1 and 5, and the breadth between toes 2 and 4 was used to characterize IT. Moreover, the letter N indicates the measurements taken from the ipsilateral side, while the letter E signifies the measurements obtained from the contralateral side. The formula of SFI is as follows:

$$SFI=-38.3\times \frac{EPL-NPL}{NPL}+109.5\times \frac{ETS-NTS}{NTS}+13.3\times \frac{EIT-NIT}{NIT}-8.8$$

### The Wet Weight Ratio of the Gastrocnemius Muscle (WWRG)

The bilateral gastrocnemius muscles were meticulously separated, and their wet weight was measured. WWRG was calculated by determining the ratio of the weight on the ipsilateral side to the weight on the contralateral side.

### Cell Culture

RSC96 cells were cultured in Dulbecco’s Modified Eagle Medium (DMEM; 10–013-CV, Corning, USA) supplemented with 10% fetal bovine serum (FBS; C0235, Invitrogen, USA). The cells were maintained at 37 °C with 5% CO_2_. NG108-15 cells were cultured in DMEM supplemented with 10% FBS and 0.1 mM hypoxanthine (H9377, Sigma-Aldrich, USA). Moreover, 400 nM aminopterin (A5159, Sigma-Aldrich, USA) and 16 μM thymidine (T9250, Sigma-Aldrich, USA) were added to induce differentiation into the neuron cell lineage. Cell passaging was performed when the cells reached 90% confluency.

### Hypoxia/Reoxygenation (H/R) Administration of NG108-15 Cells

A H/R model of NG108-15 was established to simulate the H/R environment of neurons in vitro under pathological conditions. Specifically, NG108-15 cells were seeded into a 12-well plate with a confluence degree of 30%. Following 24 h of routine culture, the growth density of the NG108-15 cells typically reached 50 to 60%. At this point, the H/R intervention was initiated. When the confluence rate of NG108-15 cells was 50 to 60%, the conventional medium in the wells was replaced with a glucose- and FBS-free medium and the cells were cultured for 6 h at 37 °C under anoxic conditions in a cell incubator with 90% N_2_, 5% CO_2_, and 5% O_2_. Subsequently, the medium was removed, and the cells were cultured with the conventional medium in a 37 °C incubator with 5% CO2 for 3 h.

### Cell Transfection

SCs were transfected with 50 nM of miR-21-5p mimic/inhibitor and their respective NCs (miR10000790-1–5; miR20004711-1–5, Ribobio Biotech, Guangzhou, China). Furthermore, for knockdown gene analysis, siRNAs targeting GAS5 and a non-targeting siRNA (si-NC) were chemically synthesized (Ribobio Biotech, Guangzhou, China). The cells were transfected with lncRNA GAS5-siRNA#1, lncRNA GAS5-siRNA#2, and lncRNA GAS5-siRNA#3, respectively. After culturing for 24–48 h, the knockdown efficiency was determined by RT-qPCR. The target sequences of these siGAS5s are shown in Table 1. All transfections were performed using lipofectamine 2000 (11668019, Invitrogen, USA) following the manufacturer’s instructions. Furthermore, RSC96 cells were infected with a lentiviral vector overexpressing GAS5 (OE-GAS5) or a lentiviral vector with an NC (OBiO Biotech, Shanghai, China) at a multiplicity of infection of 50. Cells in the control group were treated with PBS only.

**Table 1:** Target sequences of siGAS5

Genes	Primer sequence
Lnc-siGAS5-1	Sense (5′-GAUGGAUGCUUGAACAGAATT-3′)
	Antisense (5′-UUCUGUUCAAGCAUCCAUCTT-3′)
Lnc-siGAS5-2	Sense (5′-GACAUUGUGCUGUCAAGAATT-3′)
	Antisense (5′-UUCUUGACAGCACAAUGUCTT-3′)
Lnc-siGAS5-3	Sense (5′-CAAAGAUGGAUGAAAGCUATT-3′)
	Antisense (5′-UAGCUUUCAUCCAUCUUUGTT-3′)

### Cell Co-culture

After 48 h of transfection, the RSC96 cells were digested and centrifuged to obtain a 200 μL single cell suspension. This suspension was then inoculated into the upper chamber of the Transwell (0.4 μM, CLS3401, Corning, USA) at a density of 10^6^ cells/mL. NG108-15 cells in the lower chamber were subjected to the H/R administration. The co-culture was carried out at 37 °C with 5% CO_2_ for 24 h.

### Transwell Migration Assay

RSC96 cells following completion of transfection were inoculated in the upper chamber (8 μM, CLS3428, Corning, USA) as previously mentioned. In the lower chamber, 500 μL of DMEM medium containing 10% FBS was added. The co-culture was then carried out at 37 °C with 5% CO_2_ for 12 h. After the co-culture, the cells at the bottom of the upper chamber were wiped away using a cotton swab. The remaining cells were stained with 0.1% crystal violet and imaged. The cell count was performed under a microscope.

### Cell Counting Kit-8 (CCK-8)

RSC96 cells were seeded onto 96-well plates at an appropriate density (appropriately 30%). After 48 h of transfection, 10 μL of CCK-8 reagent (40203ES60, YEASEN Biotech, Shanghai, China) was injected into each well, followed by the inoculation of cells for 2 h at 37 °C with 5% CO_2_. To measure cell viability, the absorbance at 450 nm was detected using a microplate reader (Synergy 2, BioTek, USA).

### The Terminal Deoxynucleotidyl Transferase dUTP Nick End Labeling (TUNEL) Staining

At 48 h after transfection of RSC96 cells, the cells were fixed for 30 min on ice. After washing with PBS, the cells were incubated with 0.3% Triton X-100 (30188928, Sinopharm Chemical Reagent Co., Ltd, Shanghai, China) for 5 min at room temperature. Next, 50 μL of TUNEL (C1089, Beyotime, Shanghai, China) reaction buffer was added to the RSC96 cells and incubated for 1 h at 37 °C in the dark. Nuclei were stained with Hoechst 33342. To calculate the apoptotic index, the number of TUNEL-positive cells was counted and divided by the total number of cells.

### Real-Time Quantitative PCR (RT-qPCR)

For the extraction of total RNA, the Trizol reagent (Sangon Biotech, Shanghai, China) was used. The miRcute Plus miRNA First-Strand cDNA Kit (KR211-02, Tiangen Biotech, Beijing, China) was utilized to synthesize first-strand cDNA for miRNA. The miRcute Plus miRNA qPCR Kit (FP411-01, Tiangen Biotech, Beijing, China) was used for the quantification of qPCR. For GAS5, the total RNA was extracted using the same method as mentioned above. The FastKing gDNA Dispelling RT SuperMix (KR118, Tiangen Biotech, Beijing, China) was used for the synthesized first-strand cDNA. The Hieff® qPCR SYBR Green Master Mix (High Rox Plus) (11203ES08, YEASEN Biotech, Shanghai, China) was used for RT-qPCR. The primer sequences for miR-21-5p/U6 and GAS5/GAPDH are provided in Table 2. The amplification reaction conditions should be followed as per the instructions. The RT-qPCR data were analyzed using the 2^−ΔΔCT^ method.

**Table 2:** Primer sequence list

Genes	Primer sequence
miR-21-5p	GTAGCTTATCAGACTGATGTTGA
U6	CTCGCTTCGGCAGCACA
GAS5	Forward: 5′-GCAAGCCTAACTCAAGCCATTG-3′
	Reverse: 5′-CTTGCTCCACACAGTGTAGTC-3′
GAPDH	Forward: 5′-ATGACTCTACCCACGGCAAG-3′
	Reverse: 5′-GGAAGATGGTGATGGGTTTC-3′

### Western Blotting

To extract the proteins from SCs and NG108-15 cells, the whole protein extraction kit (BC3710, Solarbio, Beijing, China) was used. The protein lysates obtained were quantified using a BCA protein assay kit (P0010, Beyotime, Shanghai, China) according to the manufacturer’s instructions. Subsequently, 12.5% SDS-PAGE (P2013, NCM Biotech, Suzhou, China) was used to separate the protein samples. The separated proteins were then electrophoretically transferred onto a polyvinylidene difluoride (PVDF) membrane (IPVH00010, Millipore, USA). The membrane was incubated with 5% skim milk at room temperature for 1 h. Primary antibodies, including anti-Bcl-2, anti-Bax, anti-cleaved caspase-3 (C-cas3), and anti-GAPDH, were then added and incubated overnight at 4 °C. The secondary antibodies (anti-rabbit or anti-mouse) were then incubated at room temperature for 2 h. Detailed information of the antibodies was presented in Table 3. Protein bands were detected using an enhanced chemiluminescence system and a Bio-Spectrum Gel Imaging System (4500F, Tanon, Shanghai, China). GAPDH was used as an internal control. The gray values of the protein bands were analyzed using ImageJ software.

**Table 3:** Antibody list

Antibody	Concentration	Catalog No. R	Supplier
Rabbit anti-NF200	1:200	N4142	Sigma-Aldrich
Rabbit anti-MBP	1:200	ab40390	Abcam
Rabbit anti-βIII tubulin	1:200	ab18207	Abcam
Rabbit anti-Bcl-2	1:1000	ab59348	Abcam
Rabbit anti-Bax	1:1000	2772	CST
Rabbit anti-cleaved caspase-3	1:1000	9664	CST
Mouse anti-GAPDH	1:1000	60,004	Proteintech
Anti-rabbit HRP	1:1000	7074	CST
Anti-mouse HRP	1:1000	7076	CST
Anti-mouse antibody Alexa 555	1:200	A-21428	Thermo
Anti-rabbit antibody Alexa 488	1:800	A-11034	Thermo
Anti-mouse antibody Alexa 488	1:800	A-11001	Thermo

### Luciferase Reporter Assay

The pmir-GLO-GAS5-Wt luciferase vector and pmir-GLO-GAS5-Mut luciferase vector (Ribobio Biotech, Guangzhou, China) were constructed. Subsequently, the pmir-GLO-GAS5-Wt/Mut vectors were co-transfected into RSC96 cells with miR-21-5p mimics/mimic NC using lipofectamine 2000 according to the manufacturer’s instructions. Following 48-h incubation period, the luciferase reporter assay system was deployed to evaluate the outcomes.

### RNA Fractionation

Separation of nuclear and cytoplasmic fractions of NG-108 followed by RNA isolation was carried out using PARISTM Kit (AM1921, Invitrogen, USA). Briefly, the cultured NG-108 cells were washed with 1 × PBS and then lysed in an ice-cold cell fractionation buffer. After incubating on ice for 10 min, the lysate was centrifuged at 1000 × g for 10 min at 4 °C to separate the nuclear and cytoplasmic fractions. Following the manufacturer’s instructions, the total RNA of each particle was extracted. Various gene/transcript expression levels in both nuclear and cytoplasmic fractions of all samples were quantified by quantitative RT-qPCR as described above.

### Immunofluorescence Staining

NG108-15 cells were fixed with ice-cold 4% paraformaldehyde (PFA) and frozen sections of the sciatic nerve were permeated with 0.5% Triton X-100. To block non-specific binding, goat serum was utilized. The primary antibodies were then incubated overnight at 4 °C in appropriate proportions. β-III tubulin, a primary antibody, was employed to stain the cytoskeleton of NG108-15 cells. Neurofilament 200 (NF200) and myelin basic protein (MBP) were used to stain the axons and myelin sheath of the sciatic nerve. The secondary antibody was blocked with the same species as the primary antibody. Detailed information of the antibodies was presented in Table 3. Subsequently, fluorescence inverted microscope was employed to observe and capture images as soon as possible, followed by sealing with an anti-fluorescence quench agent. Statistical analysis of the obtained images was performed using ImageJ software.

### Statistical Analysis

Statistical analyses were conducted using GraphPad Prism 9 software. All the data are presented as the mean ± standard deviation (SD). The intergroup difference was tested by one-way analysis of variance (ANOVA) followed by Scheffe’s post hoc multiple-comparison test. Two-way ANOVA analysis was utilized to determine the impact of GAS5 and miR-21 on SCs, and neuronal neurite growth followed by Tukey’s post hoc multiple-comparison test. *P* < 0.05 was considered to be statistically significant.

## Results

### Electroacupuncture Promotes Nerve Function Recovery and Regeneration After Sciatic Nerve Injury and Induces Differential Expression of GAS5 and miR-21

**Fig. 1:**
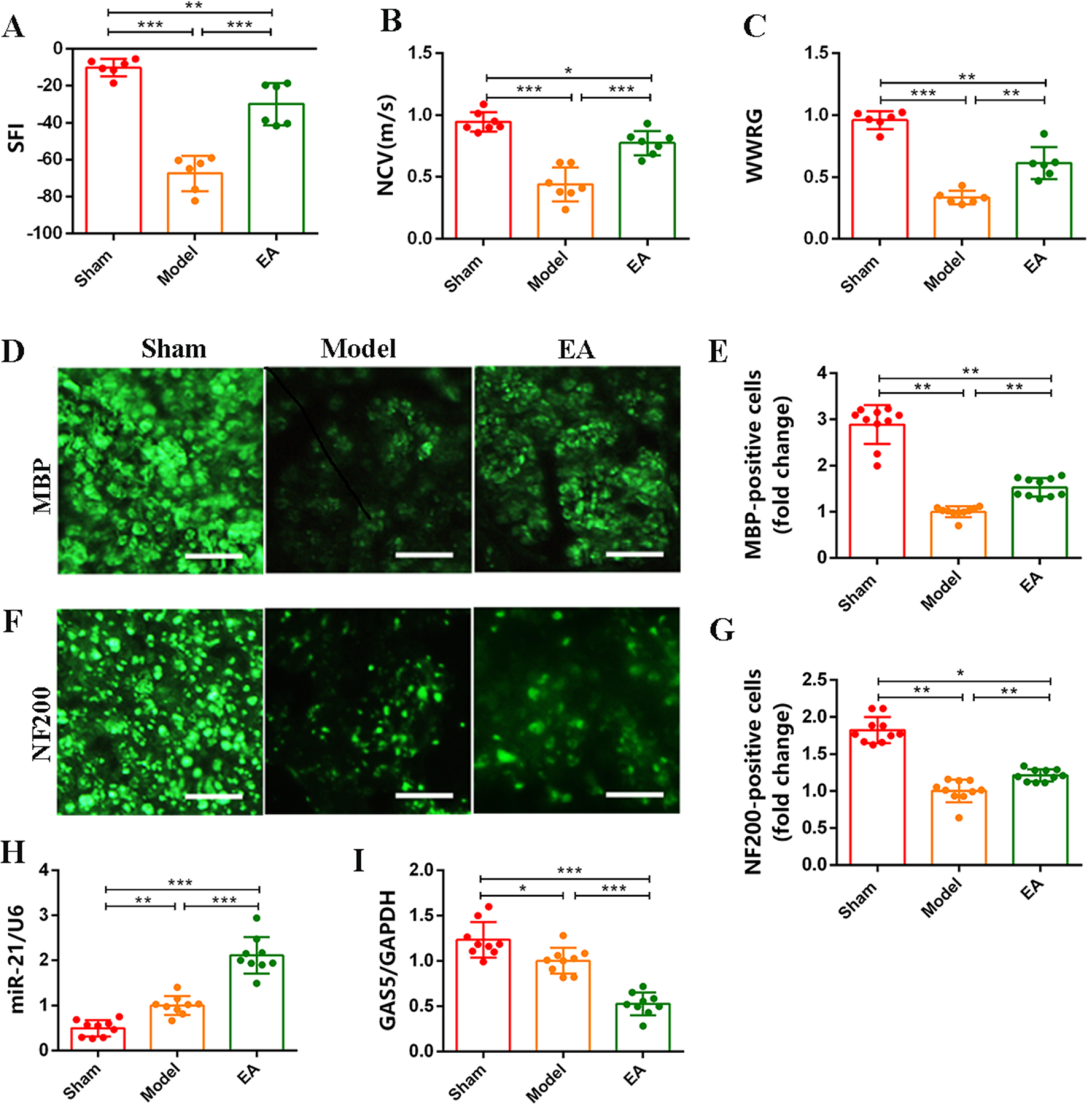
Electroacupuncture promotes nerve function recovery and regeneration after sciatic nerve injury and induces differential expression of GAS5 and miR-21. **A**–**C** The nerve function recovery index detection of SFI, NCV, and WWRG of rats. **D**, **F** Immunofluorescence staining of sciatic nerve reflected the morphology of myelin (MBP, green) and axons (NF200, green). Scale bar = 50 μm. **E**, **G** The statistical analysis of the number of myelin and axons. **H** RT-qPCR was used to detect the expression of GAS5 in the sciatic nerve. **I** RT-qPCR was used to detect the level of miR-21 in the sciatic nerve. For the above, data are represented as mean ± SD (one-way ANOVA, Scheffe’s post hoc test: *P < 0.05, **P < 0.01, ***P < 0.001)

The effect of EA on functional recovery and axon regeneration following SNI remains to be further verified. First, the behavioral assays were used to assess the functional recovery of the sciatic nerve. The results indicated a significant improvement in sciatic functional index (SFI) (Fig. 1A), nerve conductive velocity (NCV) (Fig. 1B), and wet weight ratio of the gastrocnemius (WWRG) (Fig. 1C) following EA intervention. Subsequently, the immunofluorescence staining of NF200 and MBP was performed to evaluate the effects of EA on axon and myelin regeneration after SNI. The findings revealed a substantial increase in the number of regenerated axons (Fig. 1D, E), along with an increase in the number of myelin sheaths increased and improved structure following EA intervention (Fig. 1F, G). Moreover, to further investigate the underlying repair mechanism of EA following nerve injury, RT-qPCR analysis was conducted. The results demonstrated a significant upregulation of miR-21 expression (Fig. 1H) and a significant downregulation of GAS5 expression after EA intervention (Fig. 1I). Collectively, these findings not only confirmed the reparative effects of EA after SNI but also suggested the potential involvement of differential expression of GAS5 and miR-21 in mediating the therapeutic effects of EA intervention.

### *miR-21 Mimic Promote Proliferation and Migration and Suppresses Apoptosis of SCs *In Vitro

**Fig. 2:**
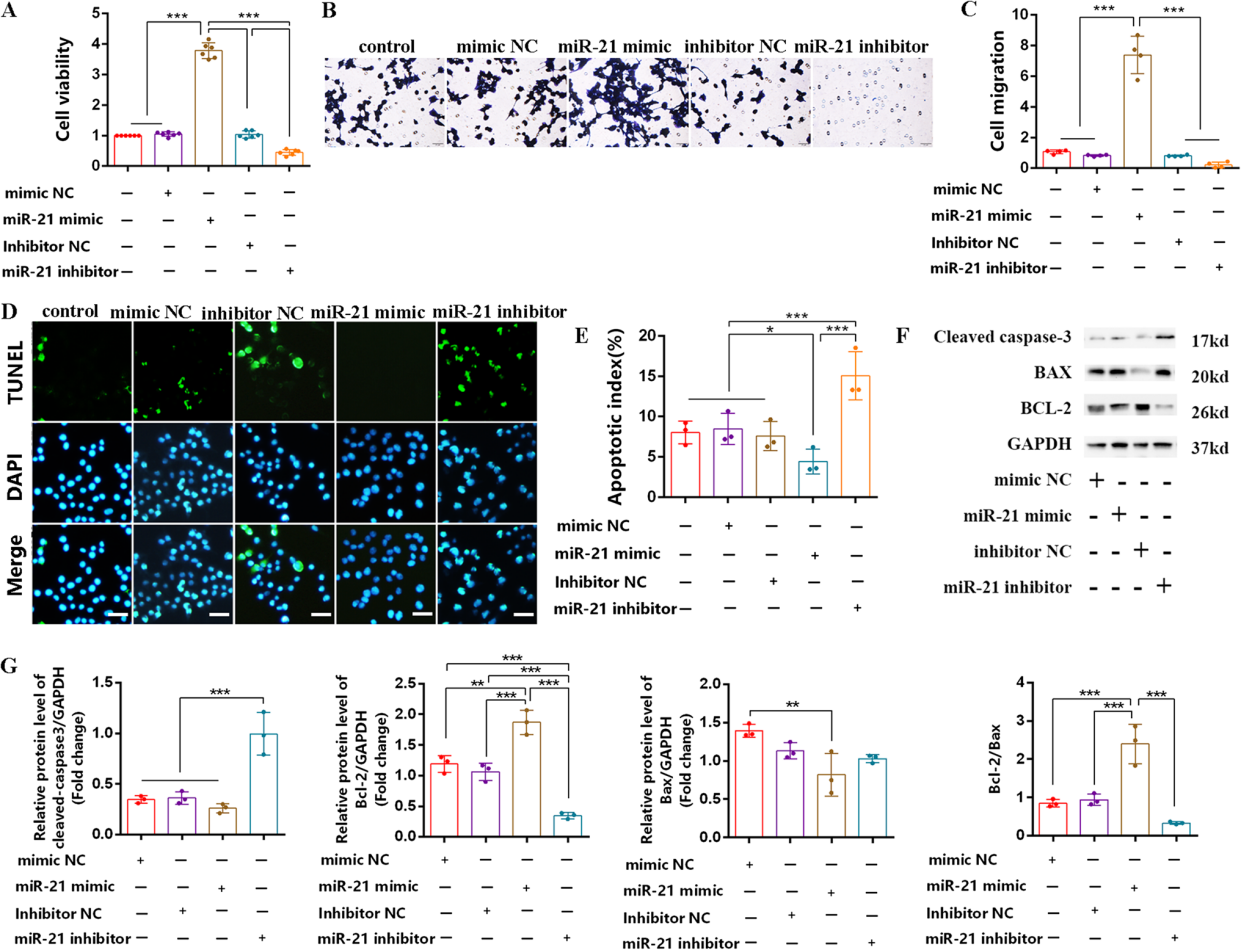
miR-21 mimic promotes proliferation and migration and suppresses apoptosis of SCs in vitro. **A** CCK-8 assay was used to examine the proliferation of SCs. **B** Crystal violet staining was used to trace the migration ability of SCs. **C** The number of cell migration statistics analysis. **D** TUNEL assay was used to detect the number of apoptotic SCs (green). Scale bar = 50 μm. **E** The statistics analysis apoptosis index of SCs. **F** Western blot was used to detect the expression of cleaved caspase-3/Bcl-2/BAX and GAPDH. **G** Statistics analysis of gray value for protein bands. For the above, data are represented as mean ± SD (one-way ANOVA, Scheffe’s post hoc test: *P < 0.05, **P < 0.01, ***P < 0.001)

To explore the potential role of miR-21 in SCs in vivo, we transfected RSC96 (rat SCs line) cells separately with miR-21 mimic, miR-21 inhibitor, and their respective negative controls (NCs). Cell Counting Kit-8 (CCK-8) and Transwell assays were performed to assess cell proliferation and migration. The results demonstrated that the miR-21 mimic significantly promoted the proliferation and migration of SCs compared to the miR-21 inhibitor (Fig. 2A–C). Additionally, terminal deoxynucleotidyl transferase dUTP nick end labeling (TUNEL) staining revealed that the miR-21 mimic led to a significant decrease in the number of apoptotic SCs, while the miR-21 inhibitor had the opposite effect (Fig. 2D, E). Results of western blot further showed that the miR-21 mimic reduced the expression of the pro-apoptotic protein cleaved caspase-3, increased the expression of the anti-apoptotic protein Bcl-2, and subsequently enhanced the Bcl-2/Bax ratio compared to the mimic NC. In contrast, the miR-21 inhibitor exhibited contrasting effects (Fig. 2F, G). Accordingly, these findings indicated that miR-21 may exert beneficial effects on SCs and contribute to the improvement of the nerve injury repair process.

### *Silencing GAS5 Promotes SCs Proliferation and Migration and Suppresses SC Apoptosis *In Vitro

**Fig. 3:**
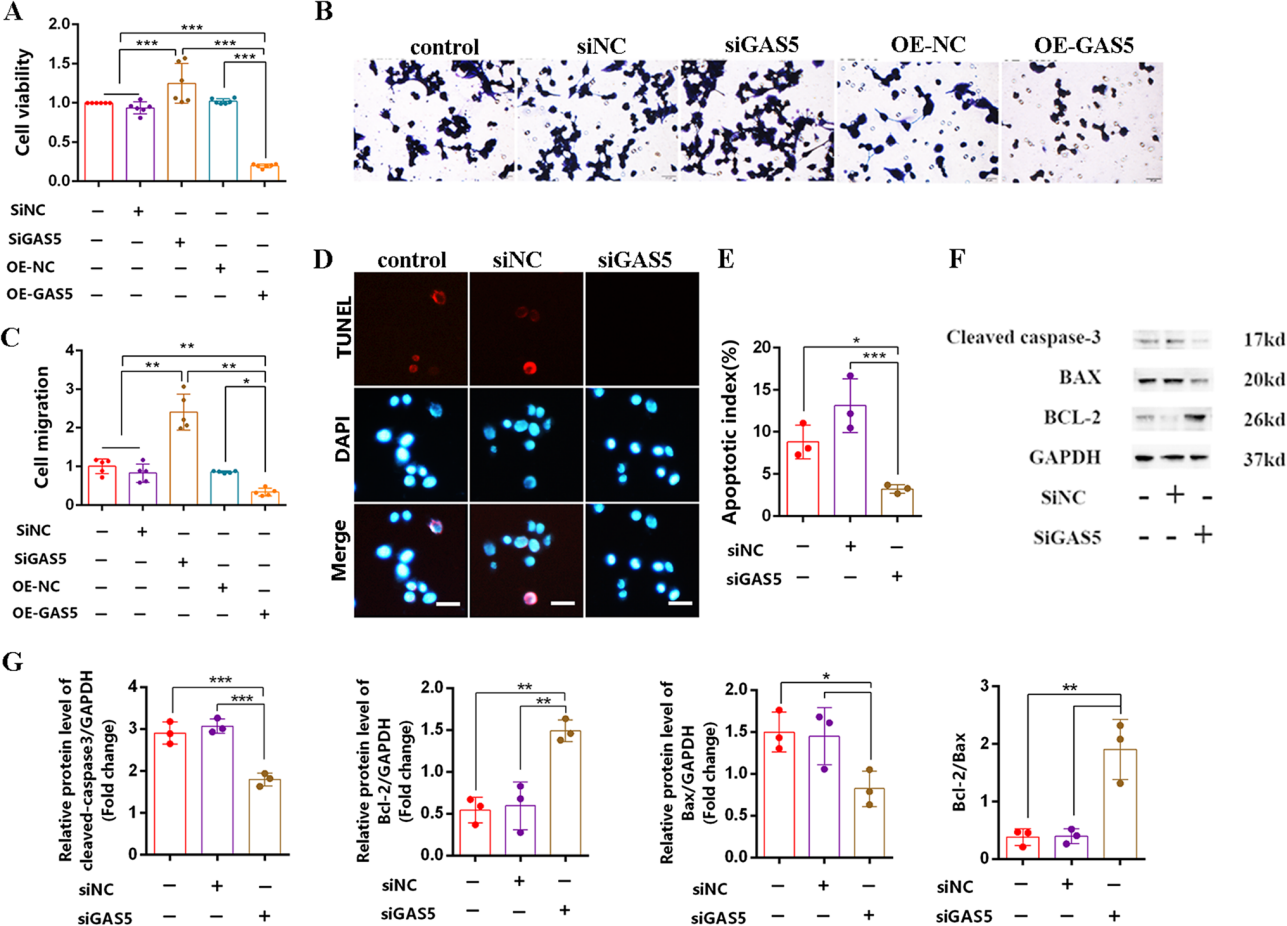
Silencing of GAS5 promotes SCs proliferation and migration and suppresses SC apoptosis in vitro. **A** CCK-8 assay was used to examine the proliferation of SCs. **B** Crystal violet staining was used to trace the migration ability of SCs. **C** The number of cell migration statistics analysis. **D** TUNEL assay was used to detect the number of apoptotic SCs (red). Scale bar = 50 μm. **E** The statistics analysis apoptosis index of SCs. **F** Western blot was used to detect the expression of cleaved caspase-3/Bcl-2/BAX and GAPDH. **G** Statistics analysis of gray value for protein bands. For the above, data are represented as mean ± SD (one-way ANOVA, Scheffe’s post hoc test: *P < 0.05, **P < 0.01, ***P < 0.001)

To further investigate the role of GAS5 in SCs, we constructed the chemically synthesized double-stranded small interfering RNA (siRNA) that knockdown GAS5 (siGAS5), and the lentiviral vector that overexpresses GAS5 (OE-GAS5). Following transfection/infection of SCs in vitro, the CCK-8 assay and Transwell assay revealed that silencing of GAS5 significantly promoted the proliferation and migration of SCs compared to the overexpression of GAS5 (Fig. 3A–C). Furthermore, using TUNEL staining, we observed a significant decrease in the number of apoptotic SCs with siGAS5 treatment (Fig. 3D, E). In addition, we examined the expression of Bcl-2/Bax and cleaved caspase-3 proteins, key regulators of cell apoptosis. Western blot analysis demonstrated that the knockdown of GAS5 downregulated the pro-apoptotic protein cleaved caspase-3 and upregulated the anti-apoptotic protein Bcl-2, resulting in an improved Bcl-2/Bax ratio and reduced the apoptosis in SCs (Fig. 3F, G). Collectively, these findings indicate that GAS5 is not conducive to SCs and may serve as an inhibitory factor in the nerve injury repair process.

### Overexpression of GAS5 Prevented the Effect of miR-21 Mimic on SC Proliferation, Migration, and Apoptosis

**Fig. 4:**
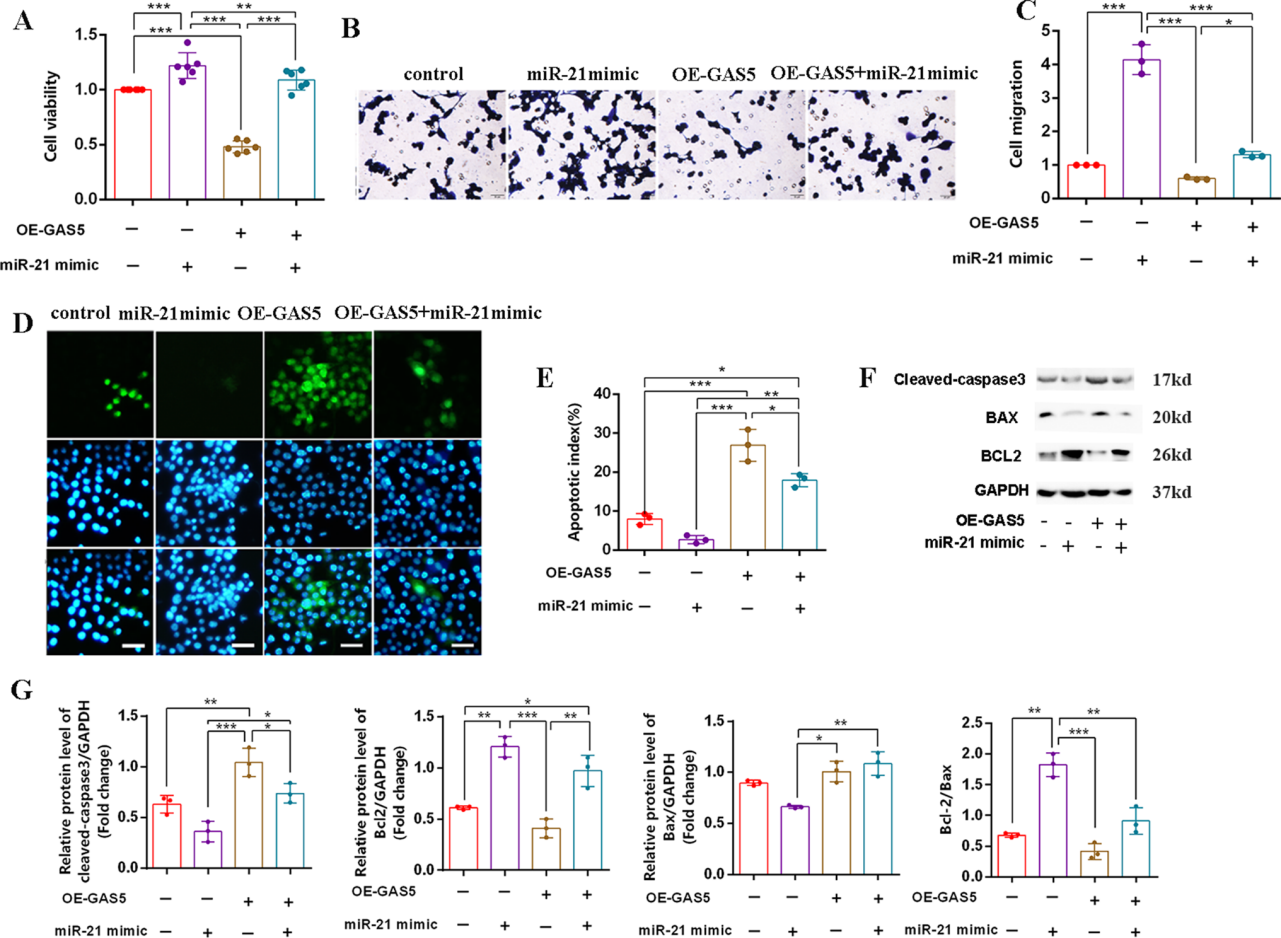
Overexpression of GAS5 prevented the effect of miR-21 mimic on SCs proliferation, migration, and apoptosis. **A** CCK-8 assay was used to examine the proliferation of SCs. **B** Crystal violet staining was used to trace the migration ability of SCs. **C** The number of cell migration statistics analysis. **D** TUNEL assay was used to detect the number of apoptotic SCs (green). Scale bar = 50 μm. **E** The statistics analysis apoptosis index of SCs. **F** Western blot was used to detect the expression of cleaved caspase-3/Bcl-2/BAX and GAPDH. **G** Statistics analysis of gray value for protein bands. For the above, data are represented as mean ± SD (two-way ANOVA, Tukey’s post hoc test: *P < 0.05, **P < 0.01, ***P < 0.001)

To elucidate the interaction between GAS5 and miR-21 in SC, the miR-21 mimic was transferred into stable lentivirus SCs strains that overexpressed GAS5. In comparison to the control group, the miR-21 mimic resulted in enhanced proliferation and migration of SCs. However, when OE-GAS5 was introduced, it reversed the effects of the miR-21 mimic on SCs (Fig. 4A–C). The TUNEL assay demonstrated that the miR-21 mimic decrease the number of apoptotic cells, while OE-GAS5 attenuated this effect (Fig. 4D, E). Western blot analysis revealed that OE-GAS5 prevented the impact of miR-21 mimic, leading to the downregulation of Bcl-2/Bax and the upregulation of cleaved caspase-3 (Fig. 4F, G). Based on these findings, it can be inferred that there may exist a potential targeting relationship between GAS5 and miR-21, which may play a role in the pathological process of PNI.

### SCs Transfected miR-21 Mimic Promoted Neurite Growth of Hypoxia/Reoxygenation (H/R)-Induced NG-108 Neurons, While Overexpression GAS5 Reversed the Effect

**Fig. 5:**
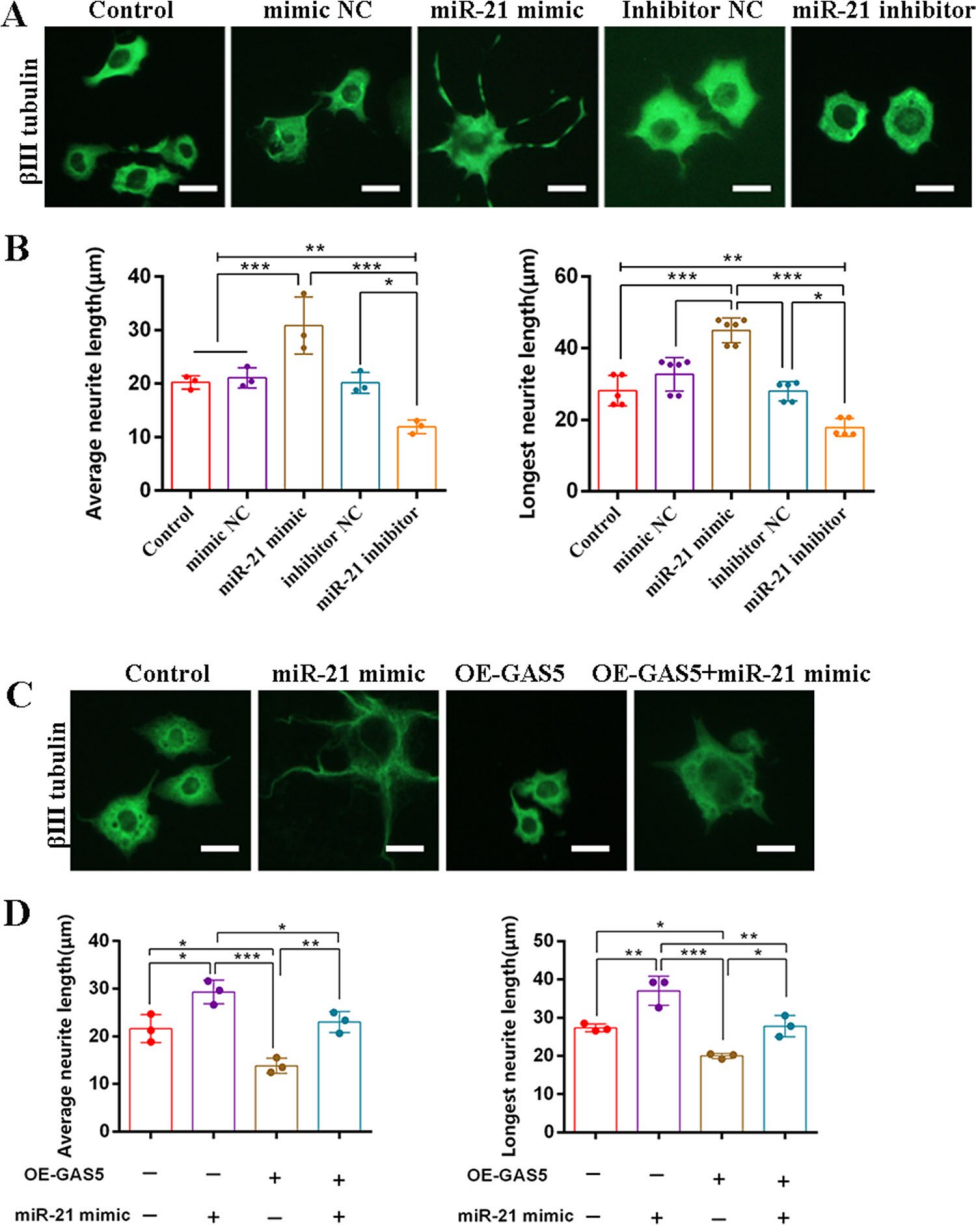
SCs transfected miR-21 mimic promoted neurite growth of H/R-induced NG-108 neurons, while OE-GAS5 reversed the effect. **A**, **C** Immunocytochemistry staining of beta III tubulin (green) to observe neurite outgrowth of NG108-15 cells. Scale bar = 25 μm. **B**, **D** Neurite length quantification and analysis containing the longest and average neurite length of NG108-15 cells. For the above, data are represented as mean ± SD (two-way ANOVA, Tukey’s post hoc test: *P < 0.05, **P < 0.01, ***P < 0.001)

To investigate the impact of GAS5 and miR-21 on neurite outgrowth in neurons, we utilized the H/R damage model. Immunofluorescence staining demonstrated that the overexpression of miR-21 significantly enhanced the neurite growth of H/R-induced neurons compared to the miR-21 inhibitor group (Fig. 5A, B). Conversely, overexpression of GAS5 significantly inhibited neurite outgrowth. Notably, the overexpression of GAS5 reversed the promoting effect of miR-21 mimic on neurite outgrowth to some extent (Fig. 5C, D). These findings support that GAS5 and miR-21 potentially interact with the neurite growth in neurons.

### GAS5 Negatively Regulates miR-21 Expression, and miR-21 Is the Direct Target of GAS5

**Fig. 6:**
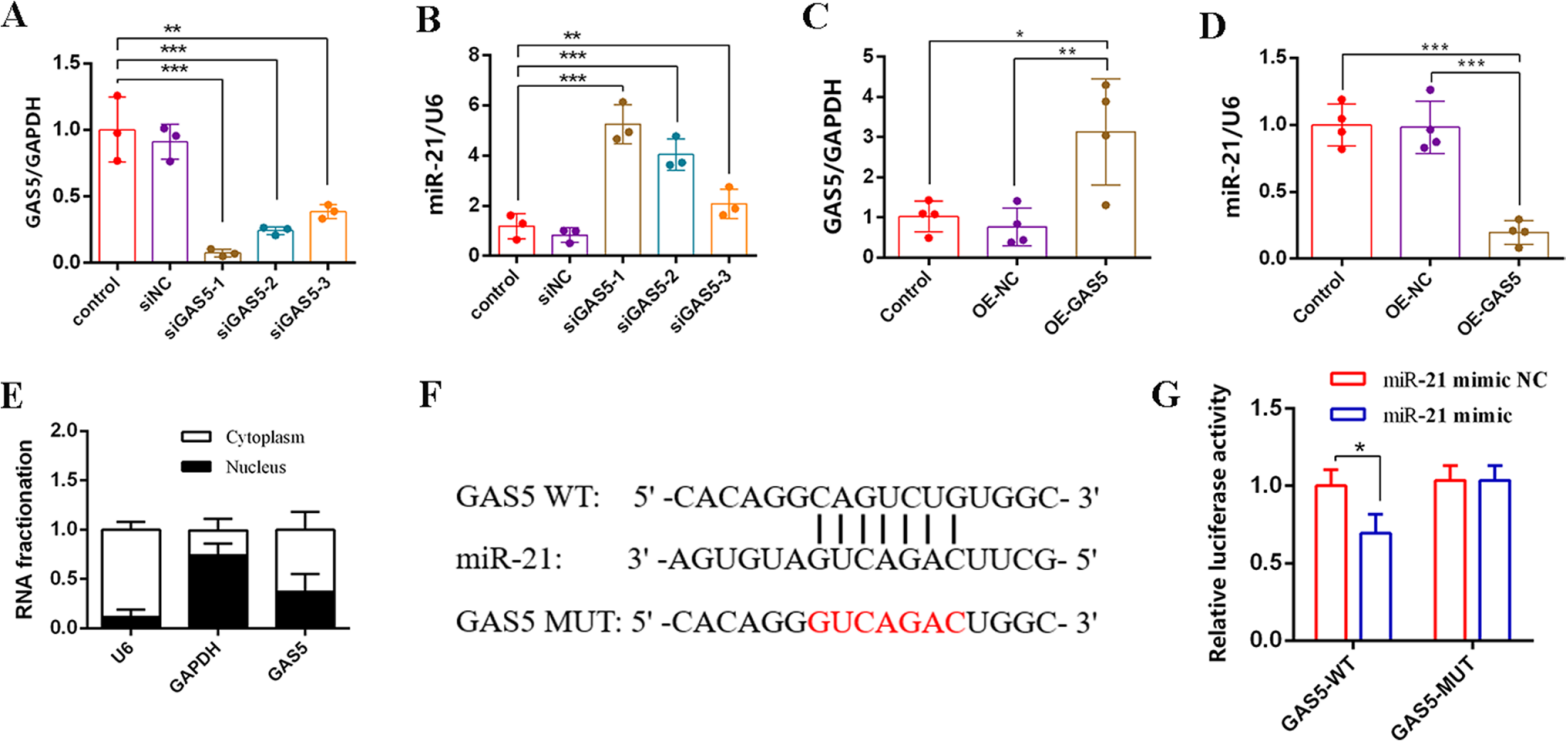
GAS5 negatively regulates miR-21 expression, and miR-21 is the direct target of GAS5. **A** RT-qPCR was used to detect the expression of GAS5 with different siRNA segments in SCs. **B** RT-qPCR was used to detect the level of miR-21 with different knocking efficiency of GAS5 in SCs. **C** RT-qPCR was used to detect the expression of GAS5 when infected lentivirus expression of GAS5 in SCs. **D** RT-qPCR was used to detect the expression of miR-21 when infected lentivirus that expression of GAS5 in SCs. **E** Subcellular fractionation assay measured the localization of GAS5 in SCs. **F** The binding sites between GAS5 and miR-21 are depicted in this diagram. **G** The dual-luciferase reporter gene assay was used to detect the luciferase activity of GAS5 when SCs transfected miR-21 mimic or NC. For the above, data are represented as mean ± SD (one-way ANOVA, Scheffe’s post hoc test: *P < 0.05, **P < 0.01, ***P < 0.001)

To investigate the potential role of GAS5 in regulating miR-21 and nerve repair, we measured miR-21 levels in SCs transfected with a siGAS5 or LV-GAS5 using RT-qPCR. Firstly, three specific siRNAs targeting GAS5 were used to assess the knockdown efficiency of GAS5 knockdown in RSC96 cells. The results revealed that all three siRNAs decreased the expression of GAS5, leading to a simultaneous upregulation of miR-21 expression (Fig. 6A, B). Among the siRNAs, the si-lnc-GAS5-3 demonstrated better efficiency and was selected for further experiments. On the other hand, the LV-GAS5 infection in SCs significantly increased the expression of GAS5, while the level of miR-21 was downregulated (Fig. 6C, D). To gain further insight into the mechanism of GAS5, it is necessary to determine the subcellular localization of GAS5. RNA fractionation results showed that GAS5 was predominantly located in the cytoplasm (Fig. 6E), suggesting it may act as a miRNA sponge. Additionally, potential binding sites between GAS5 and miR-21 were identified (Fig. 6F). Notably, the dual-luciferase reporter gene assay demonstrated that the miR-21 mimic had a significant impact on the luciferase activity of wild-type GAS5 (Fig. 6G). In summary, these experimental data demonstrated that miR-21 is the direct target of GAS5.

### Effect of EA on Nerve Regeneration and Functional Recovery Following Sciatic Nerve Injury Is GAS5-Dependent

**Fig. 7:**
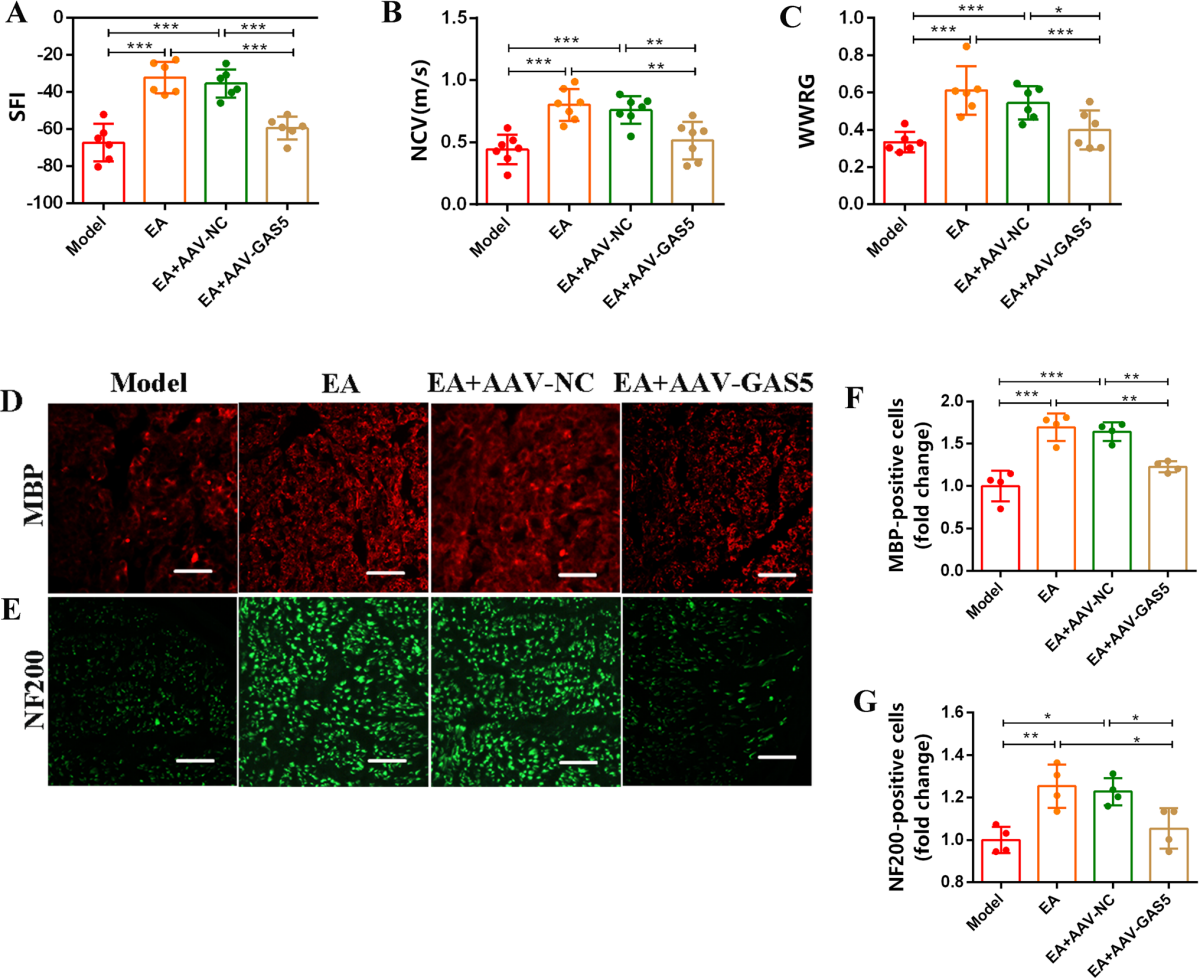
Effect of EA on nerve regeneration and functional recovery after sciatic nerve injury is GAS5-dependent. **A**–**C** The nerve function recovery index detection of SFI, NCV, and WWRG of the SNI rats. **D**, **F** Immunofluorescence staining of the sciatic nerve reflected the morphology of myelin (MBP, red) and axons (NF200, green). Scale bar = 50 μm. **E**, **G** Statistical analysis of the number of myelin and axons in the sciatic nerve. For the above, data are represented as mean ± SD (one-way ANOVA, Scheffe’s post hoc test: *P < 0.05, **P < 0.01, ***P < 0.001)

To further confirm the role of GAS5 in the repair of SNI induced by EA, the adeno-associated viruses overexpressing GAS5 (AAV-GAS5) were injected into the injured area of SNI rats simultaneously treated with EA. The behavioral assay data revealed that the injection of AAV-GAS5 effectively reversed the function recovery of EA compared to both the EA group and the EA + AAV-NC group (Fig. 7A–C). Additionally, the immunofluorescence staining demonstrated that nerve regeneration was impeded upon the administration of AAV-GAS5 (Fig. 7D–G). Collectively, our findings provide compelling and direct evidence supporting the involvement of GAS5 participates in the EA-induced of SNI repair.

## Discussion

PNI is a common disease in the nervous system, which significantly impairs the life quality of patients [29]. Despite ongoing efforts, the repair and treatment of SNI is still a problem today [30]. Acupoint stimulation, a traditional superficial peripheral stimulation approach with a history spanning over 2500 years, has been widely utilized in clinical practice for the treatment of various diseases [31, 32]. EA, a technique that combines acupuncture with modern electrical stimulation, has shown promising results in proving dysfunctions associated with PNI [33]. Moreover, EA offers advantages such as safety, ease of use, and affordability, making it a potentially preferred therapeutic intervention for PNI. In this study, we established an SNI rat model and administered EA intervention. Consistent with the results of previous studies [34, 35], our findings demonstrated that EA promotes axonal extension, regeneration of myelin, and functional recovery following SNI. These results provide further direct evidence supporting the effectiveness of EA in the treatment of PNI. In this study, we aimed to investigate the underlying cellular and molecular mechanism of EA for nerve repair after SNI.

The PNS is primarily composed of axons of motor neurons and the myelin sheath formed by SCs. In addition to the inherent regenerative capacity of neurons, SCs create a favorable microenvironment for axon regeneration, promoting the regeneration and repair of PNI. Despite these regenerative mechanisms, the rate of nerve regeneration after PNI is often slow, leading to less satisfactory outcomes in terms of repair. Precise regulation of gene expression is essential for long-distance axon regeneration following PNI. Recent experiments have highlighted the significant impact of differentially expressed non-coding RNAs (ncRNAs), particularly miRNAs and lncRNAs on axon regeneration during PNI [36]. MiRNAs have been shown to play a role in regulating the biological behaviors of neurons and SCs [37], including neuronal survival, axonal outgrowth, and SCs phenotype [38, 39]. Among these miRNAs, miR-21 has emerged as one of the most commonly upregulated miRNAs and plays a crucial role in various cellular biological processes under physiological and pathological conditions [40]. Our findings demonstrate a significant upregulation of miRNA-21 expression following EA. According to the existing research, miR-21 promotes axon growth and facilitates nerve function recovery [41]. Notably, Strickland et al. [42] also observed a substantial enhancement in axon growth of DRG in vitro upon overexpression of miRNA-21. Collectively, these results highlight the involvement of miR-21 in nerve injury and repair processes.

Recently, it has been reported that the expression levels of certain lncRNAs changes after PNI potentially affect nerve regeneration [43, 44]. In our study, we observed a decrease in the expression of GAS5 following EA treatment. This led us to propose that EA may exert its effects by downregulating GAS5. To gain a deeper understanding of the role of GAS5 in EA treatment after PNI, we utilized overexpressed GAS5 (AAV-GAS5). The results demonstrated that AAV-GAS5 weakened the effects of EA, which further confirmed that EA promotes SNI repair through the regulation of GAS5. The mechanisms of lncRNAs are comparatively complex, involving miRNA sponges, regulation of transcription factor activity, interactions with RNA binding proteins, and so on. The mechanism of lncRNAs depends on their subcellular localization. Only lncRNAs located in the cytoplasm can function as miRNA sponge. Our results indicated that GAS5 was primarily located in the cytoplasm, suggesting a potential regulatory mechanism for GAS5. Previous studies by Zhang et al. [45] have shown that in humans, GAS5 directly interacted with the assumed binding site of miR-21 at exon 4, making it a direct target of miR-21. This study also identified that GAS5 negatively regulated miR-21, possibly through its interaction with the RNA-induced silencing complex (RISC), suggesting a reciprocal repression feedback loop between miR-21 and GAS5 [45]. Recently, numerous studies have reported a negative correlation between miR-21 and GAS5 in various diseases [46]. Based on our findings in this study, it could be inferred that EA may exert its therapeutic effects by regulating the GAS5/miR-21 axis. However, further investigation is required to elucidate the detailed cellular mechanism of the GAS5/miR-21 axis in vitro.

The activation of SCs plays a crucial role in maintaining neuron survival, guiding axon regeneration, and promoting myelin sheath formation after PNI. The quantity and functional level of SCs determine the extent of peripheral nerve regeneration and repair. Insufficient numbers of SCs are often unable to support complete nerve regeneration, leading to the formation of nerve scars. Therefore, we conducted further investigations to explore the effects of GAS5 and miR-21 on SCs in vitro. MiR-21 has previously been extensively studied for its involvement in nerve injury repair. In our study, we demonstrated that the upregulated miR-21 promotes the proliferation and migration of SCs while preventing their apoptosis. Conversely, knockdown of the miR-21 has the opposite effect. This is consistent with the findings of Ning et al. [47], who reported that miRNA-21 promoted SCs proliferation during nerve injury repair. GAS5 has been widely recognized as an inhibitor of axon growth. Interestingly, our data revealed that the knockdown of GAS5 promotes the proliferation and migration of SCs while inhibiting apoptosis. Furthermore, we found that overexpression of GAS5 reversed the effects of miR-21 upregulation on SCs. In addition, the dual-luciferase reporter assay confirmed the targeted interaction between GAS5 and miR-21. Thus, the interaction between miR-21 and GAS5 becomes more apparent. Previous studies have demonstrated that GAS5 acted as a sponge for miR-21, influencing the proliferation and migration of cancer cells [48]. Additionally, GAS5 has been shown to mediate H/R-induced cardiomyocyte apoptosis via targeting miR-21 [49]. Overall, SCs play a crucial supportive role in nerve repair after injury, and their quantity and function are essential for successful nerve regeneration and repair. Our data provided evidence that the GAS5/miR21 axis may be involved in regulating the function and activity of SCs.

In addition, enhancing the outgrowth of neuronal axons is crucial for achieving structural and functional recovery after nerve injury. NG108-15 cells, a neuronal cell line, have been extensively used in the literature [27, 50]. To simulate the neuronal injury, the H/R model was applied to NG108-15 to mimic the injury of neurons [51]. Our data demonstrated that the upregulation of miR-21 promotes neurite growth in H/R-induced NG108-15 neurons. This is consistent with the findings of Wang et al. [52], who showed that miR-21 reduced H/R-induced neuron damage and apoptosis. Other studies have also reported that miR-21 could regulate apoptosis, differentiation [53], and neurite growth of neurons [12, 54]. Furthermore, recent studies have shown that GAS5 overexpression could inhibit axon growth in DRG neurons [20]. Silencing GAS5 has been found to inhibit neuron cell apoptosis [55] and improve neurological function [19]. Our findings further revealed that overexpression of GAS5 counteracts the effect of miR-21. This is consistent with previous research demonstrating that GAS5 has a complementary region with miR-21, allowing it to suppress miR-21 expression [45]. These results suggest that GAS5 may play a regulatory role in neuronal neurite growth by modulating the activity of miR-21.

There were several limitations. Compared to cell lines in this study, primary cells might be able to more closely simulate the physiological and pathological conditions of cells, thereby providing more convincing evidence. Besides, the exploration of the target genes and signaling pathways of miR-21 has remained incomplete, warranting further investigation to enhance our understanding of its precise mechanism.

## Conclusion

In conclusion, our findings provided compelling evidence that EA, as a peripheral stimulation technique, effectively enhanced motor function and promoted nerve regeneration in rats with SNI through the regulation of GAS5. Notably, GAS5 was involved in this process by targeting miR-21. These results established a solid experimental foundation for the application of EA in the treatment of PNI, highlighting acupuncture as a promising therapeutic intervention to facilitate nerve repair following nerve injury.

## Data Availability

All data generated or analyzed during this study are available from the corresponding author’s email.
