# Correction: Electroacupuncture Promotes Nerve Regeneration and Functional Recovery Through Regulating lncRNA GAS5 Targeting miR-21 After Sciatic Nerve Injury

**DOI:** 10.1007/s12035-024-03932-z

**Published:** 2024-01-24

**Authors:** Ming-yue Tian, Yi-duo Yang, Wan-ting Qin, Bao-nian Liu, Fang-fang Mou, Jing Zhu, Hai-dong Guo, Shui-jin Shao

**Affiliations:** School of Integrative Medicine, Shanghai University of Traditional Chinese Medicine, Shanghai, 201203 China

**Correction: Molecular Neurobiology**

10.1007/s12035-023-03613-3

The original version of this article unfortunately contained error in Figure 4.

The corrected Figure 4B, D, F and the complete Figure 4 are hereby published.

**Fig. 4:**
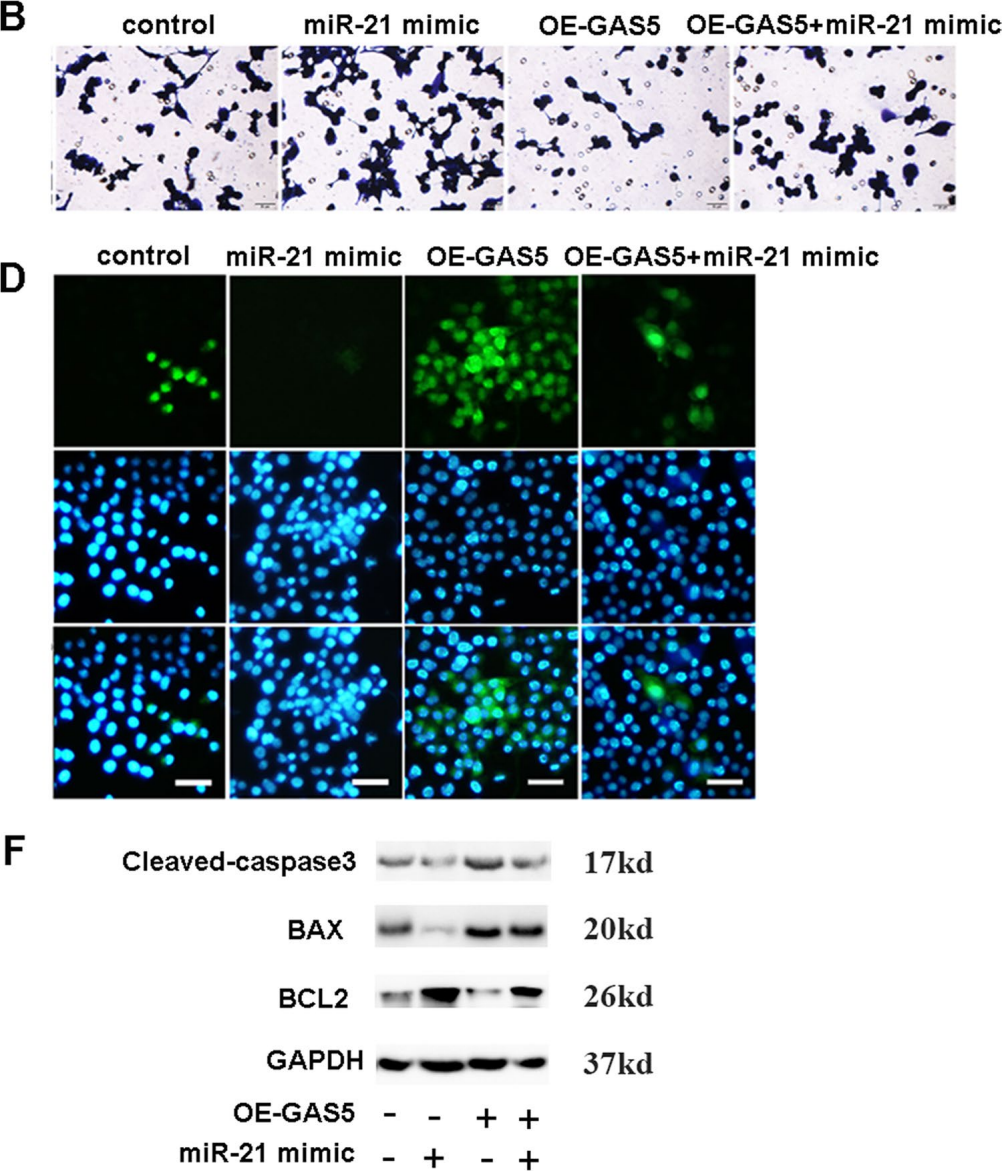
Corrected Figure 4B, D, F

**Fig. 4:**
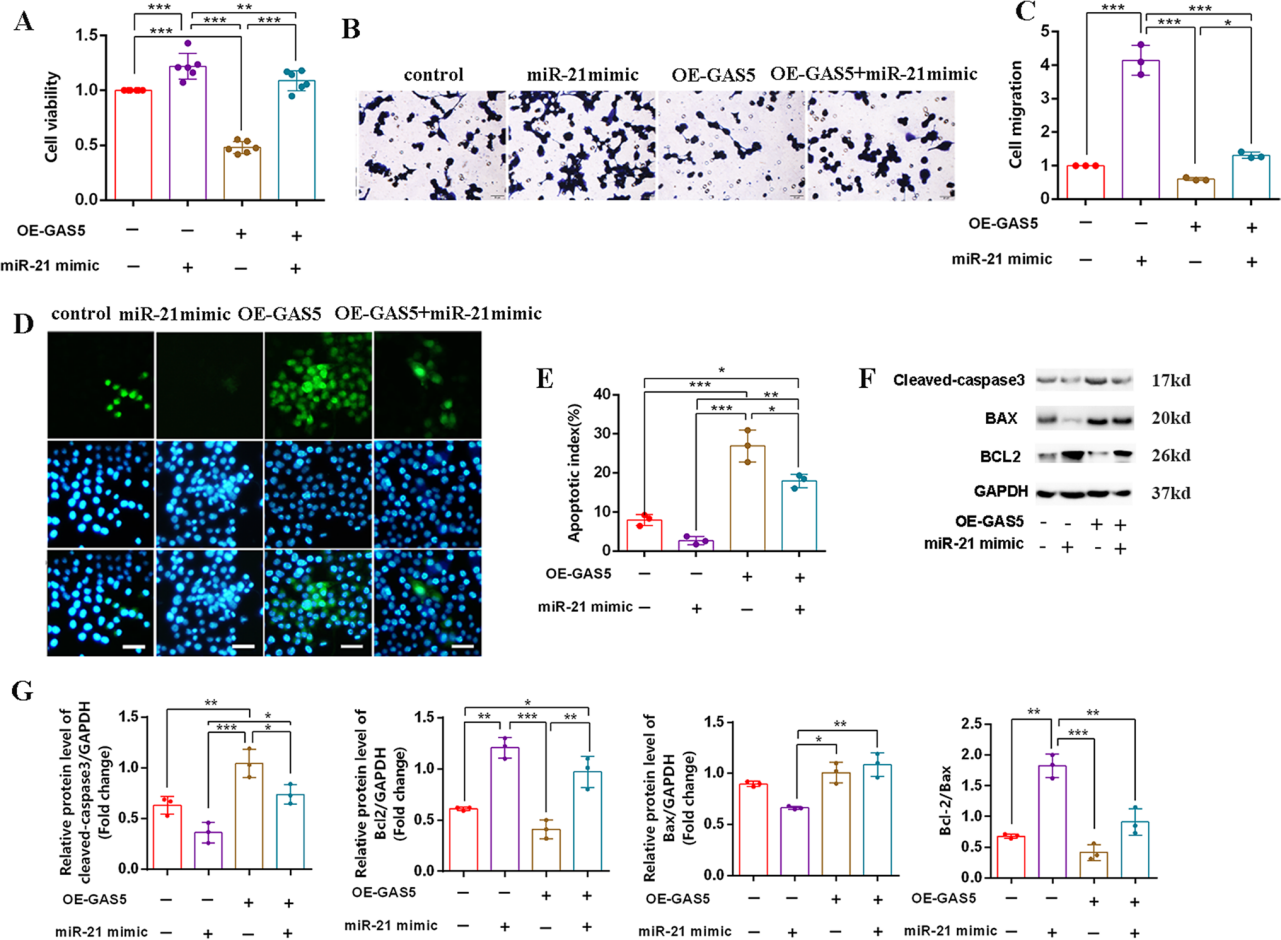
Corrected Fig. 4

The corrected Fig.4 is hereby published. The corrections made to the images in this corrigendum do not affect the legend, results, or conclusions of this study.

The original article has been corrected.

